# Small-angle scattering studies on diverse peptide-based nanotube and helical ribbon structures reveal distinct form and structure factors

**DOI:** 10.1107/S1600576725004637

**Published:** 2025-07-08

**Authors:** Ian W. Hamley, Valeria Castelletto

**Affiliations:** a School of Chemistry, Food Biosciences and Pharmacy University of Reading Whiteknights ReadingRG6 6AD United Kingdom; The University of Western Australia, Australia

**Keywords:** small-angle scattering, nanotubes, peptides, form factors, structure factors

## Abstract

Peptide-based nanotubes are bio-based self-assembled nanostructures with intriguing structural and functional properties. The structure of such nanotubes can be probed in detail using small-angle scattering experiments due to the typical length scales, *i.e.* diameter and wall thickness of the nanotubes, which span the range accessible in small-angle X-ray scattering (SAXS) or small-angle neutron scattering (SANS) studies. Here, we present SAXS data for several classes of peptide and lipopeptide systems previously studied by our group, as well as newly reported data for model short lysine-sequence lipopeptides. Previous data are re-examined using more accurate models for data plotted on Kratky plots, which emphasizes fine details of nanotube structure. In some cases, consideration of structure-factor effects is necessary to allow for the coexisting structures, and a lamellar structure factor is used to describe this. In other cases, such as several examples of surfactant-like peptides, only a form factor has to be considered to accurately fit the measured SAXS data. In these cases, a form factor for hollow nanotubes with a Gaussian bilayer profile to represent the layered peptide ordering in the nanotube walls is used to model the data. A general expression for the cross section scattering form factor is provided, which can be used for any scattering density profile (electron density for SAXS or scattering length density for SANS) across the wall. This is analysed along with the form factor for multishell (multiwall) nanotube structures with a series of slabs to represent the scattering density profile. For lipopeptides C_16_-KFK and C_16_-K (C_16_ indicates a hexa­decyl lipid chain), SAXS data show aperiodicity in the form-factor oscillations, as well as apparent broad structure-factor peaks. These features cannot be fitted using solely nanotube form-factor models, this being ascribed to the presence of coexisting structures. Lastly, for comparison, the form factors for helical ribbon and cochleate (scroll) structures are evaluated for several examples, since in many cases electron microscopy of peptide- and lipopeptide-based nanotube systems reveals the coexistence of nanotubes with such structures, related to nanotubes.

## Abstract 2

Peptide nanotubes have diverse structures, which can be elucidated by small-angle scattering. Here, we discuss in detail appropriate models for form- and structure-factor effects exemplified by analysis of data for several classes of peptide nanotubes.

## 1. Introduction

A diversity of types of peptides and lipopeptides can self-assemble in suitable solution conditions into nanotubes (Gao & Matsui, 2005 ▸; Shimizu *et al.*, 2005 ▸; Scanlon & Aggeli, 2008 ▸; Brea *et al.*, 2010 ▸; Valéry *et al.*, 2011 ▸; Chapman *et al.*, 2012 ▸; Hamley, 2014 ▸; Shimizu *et al.*, 2020 ▸; Hamley, 2020 ▸), with a range of potential applications such as biocatalysis (Huang *et al.*, 2013 ▸; Omosun *et al.*, 2017 ▸; Sarkhel *et al.*, 2020 ▸; Reja *et al.*, 2020 ▸), biomedical uses (Chen *et al.*, 2010 ▸; Lin *et al.*, 2014 ▸; Rad-Malekshahi *et al.*, 2016 ▸; Manandhar *et al.*, 2019 ▸; Hsieh & Liaw, 2019 ▸) or encapsulation/release (von Maltzahn *et al.*, 2003 ▸; Kameta *et al.*, 2011 ▸; Silva *et al.*, 2013 ▸; Wang *et al.*, 2014 ▸; Nambiar *et al.*, 2019 ▸; Sun *et al.*, 2020 ▸). Detailed studies using small-angle scattering and X-ray diffraction, among other methods, have revealed the structures of several classes of peptide nanotubes. These include wrapped β-sheet structures (Lu *et al.*, 2003 ▸; Krysmann *et al.*, 2008 ▸; Mehta *et al.*, 2008 ▸; Hamley *et al.*, 2013*a* ▸; Hamley *et al.*, 2013*b* ▸; Morris *et al.*, 2013 ▸; Madine *et al.*, 2013 ▸; Koc *et al.*, 2017 ▸; Chatterjee *et al.*, 2020 ▸; Omosun *et al.*, 2017 ▸; Reja *et al.*, 2020 ▸; Li *et al.*, 2020 ▸), stacked peptide dimer laminate structures (Valéry *et al.*, 2003 ▸; Valéry *et al.*, 2011 ▸; Tarabout *et al.*, 2011 ▸), stacked cyclic peptides with alternating d- and l- residues (Ghadiri *et al.*, 1994 ▸; Hartgerink *et al.*, 1996 ▸; Brea *et al.*, 2010 ▸), and coiled-coil peptide assemblies (Pandya *et al.*, 2000 ▸; Woolfson, 2005 ▸; Xu *et al.*, 2013 ▸; Burgess *et al.*, 2015 ▸; Wu *et al.*, 2017 ▸; Tian *et al.*, 2018 ▸). A further recently discovered class comprises nanotubes with α-helical surfactant-like peptides arranged as a bilayer perpendicular to the nanotube wall (Castelletto *et al.*, 2020 ▸; Castelletto *et al.*, 2021 ▸). Di­phenyl­alanine nanotubes are another category of extensively studied model peptide nanotubes, which have been shown to possess a remarkable range of optoelectronic, quantum and mechanical properties (Reches & Gazit, 2003 ▸; Adler-Abramovich & Gazit, 2014 ▸; Gazit, 2018 ▸; Wei *et al.*, 2017 ▸; Makam & Gazit, 2018 ▸; Ke *et al.*, 2020 ▸).

**Figure 1:**
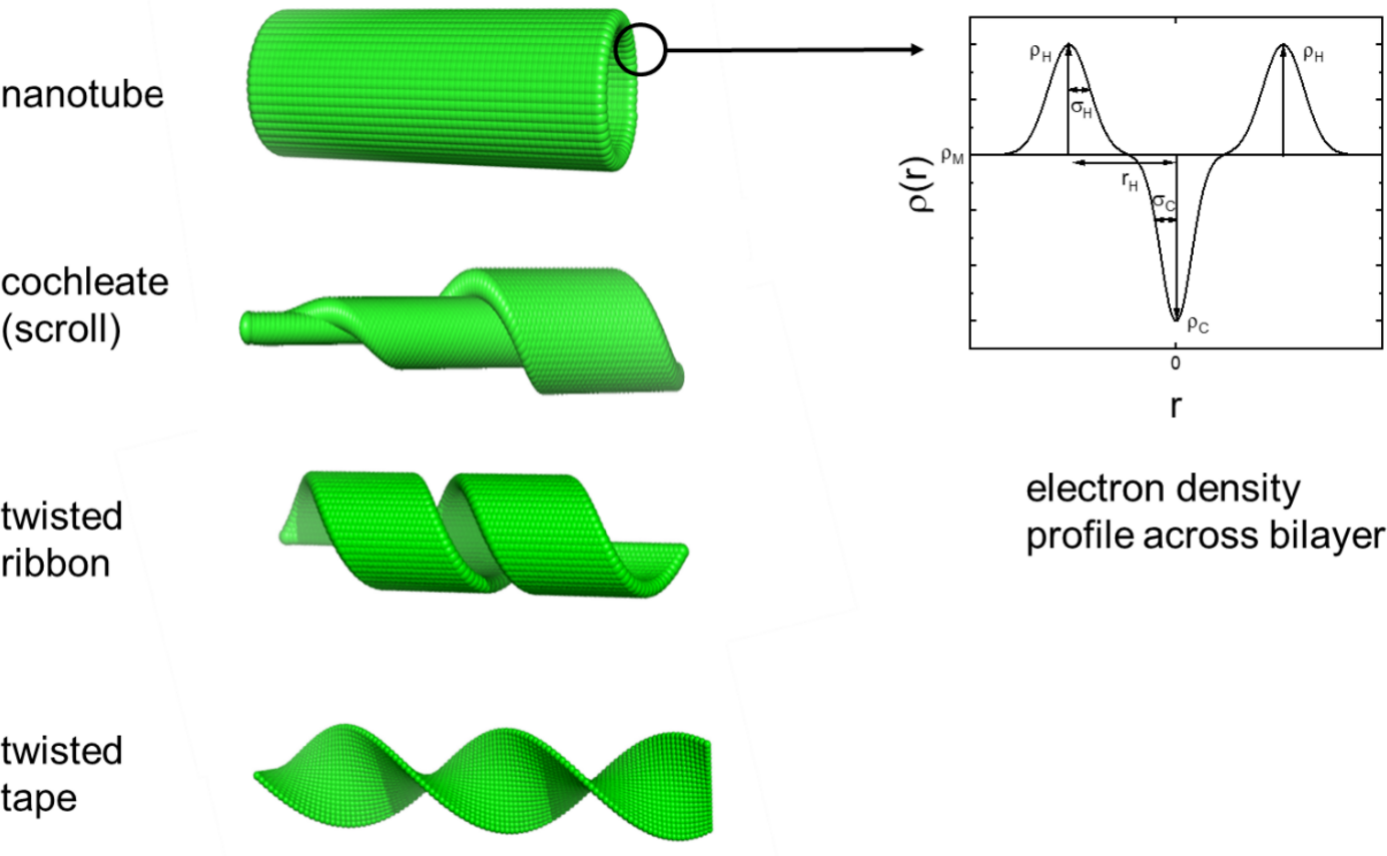
Schematic of selected lipopeptide bilayer structures along with a bilayer Gaussian electron-density profile used in form-factor modelling.

Since peptide nanotubes typically have radii in the range of tens to hundreds of nanometres and wall thicknesses of several tens of ångströms, small-angle X-ray scattering (SAXS) and small-angle neutron scattering (SANS) are excellent techniques to probe their structure. Among many examples, Lynn’s group showed that the ‘amyloid’ peptide KLVFFAE can form nanotubes under appropriate pH conditions in aqueous solution, which leads to well defined form-factor features in SAXS and SANS patterns (Lu *et al.*, 2003 ▸; Lu *et al.*, 2007 ▸). The development of a structure factor upon increasing salt concentration (especially divalent sulfate) in SAXS data was noted (Lu *et al.*, 2007 ▸). In studies on the cyclic peptide lanreotide, high-quality SAXS data were obtained that reveal nanotubes with low dispersity and narrow wall thickness. SAXS further shows that a hexagonal phase forms at high concentration (Valéry *et al.*, 2003 ▸). Time-resolved SAXS was also used to probe the lanreotide-templated silica mineralization of the nanotubes (Pouget *et al.*, 2007 ▸). It was later shown that the nanotube dimensions can be tuned by altering the peptide sequence (aromatic residue substitutions), as characterized by SAXS and electron microscopy (Tarabout *et al.*, 2011 ▸). The simple surfactant-like peptide (SLP) A_6_K (A: alanine; K: lysine) forms nanotubes at high concentration in aqueous solution, and these are present in a nematic phase due to orientational ordering of the nanotubes (Bucak *et al.*, 2009 ▸; Cenker *et al.*, 2011 ▸; Cenker *et al.*, 2012 ▸). The nanotubes are rather monodisperse in diameter, which gives well defined form-factor oscillations. The SLP forms a hexagonal phase at higher concentration (Cenker *et al.*, 2011 ▸). Siegl *et al.* (2021 ▸) have performed SAXS on multilayer nanotubes formed by layer-by-layer assembly of the cationic dimeric amino acid conjugate C_12_KC_12_K (C_12_: dodecyl; K: lysine) with anionic sodium polymethacrylate, and cryo-TEM (transmission electron microscopy) revealed multiwall nanotubes and multiwall helical ribbons. The SAXS data were fitted using a model defined by the product of the form factor of a hollow cylinder and a paracrystalline structure factor to describe the multilayer structure. A simple lipidated amino acid C_16_-K (C_16_ indicates a hexa­decyl lipid chain) forms nanotube-related cochleate (scroll) structures in aqueous salt solution or helical ribbon structures (Fig. 1 ▸) depending on pH, for which detailed SAXS measurements were performed (Gao *et al.*, 2019 ▸), including fits to a helical ribbon form factor (McCourt *et al.*, 2022 ▸). SAXS data were used to complement high-resolution cryo-TEM in the determination of the cross-β nanotube structure of Ac-FKFEFKFE-NH_2_, which forms helical ribbons and nanotubes (with coexisting nanotube morphologies) (Wang *et al.*, 2021 ▸). The SAXS profiles could be well described using profiles calculated from helical reconstruction of electron micrograph images. SAXS data have also been presented for nanotubes formed by α-helical hairpin peptides (Hughes *et al.*, 2019 ▸). In another class, tetrameric coiled-coil peptide bundles can form nanotubes dependent on pH, and the SAXS form factor was described using a core–shell cylinder form factor (Tian *et al.*, 2018 ▸). Other examples are covered in a review dedicated to SAXS and X-ray diffraction (XRD) studies of peptide nanotubes (Narayanan *et al.*, 2021*a* ▸).

Our group has elucidated the structure of nanotubes formed by a variety of peptides and lipopeptides. The amyloid peptide AAKLVFF containing the KLVFF core sequence from the amyloid β peptide forms nanotubes in methanol, the structure of which was determined from the SAXS form factor, whilst anisotropic SANS patterns were observed due to nematic ordering (Krysmann *et al.*, 2008 ▸). Later we showed that a lipopeptide containing the same KLVFF sequence in C_16_-KKFFVLK (reading from C- to N-terminus) forms rather monodisperse nanotubes in aqueous solution (and also gives a well defined helical XRD pattern, resulting from the bilayer wrapping in the nanotube wall) (Hamley *et al.*, 2013*b* ▸). The SAXS data are discussed in more detail below, along with an improved fit which also includes a structure factor. Another notable feature of this system is that the lipopeptide tubes/ribbons show thermoreversible unwrapping into helical ribbons (also shown by cryo-TEM). We have investigated lipopeptides containing bioactive sequences such as the RGDS integrin-binding motif, and lipopeptide C_14_-WGGRGDS forms nanotubes coexisting with right-handed helical ribbons (0.1 wt% PBS solution) (Rosa *et al.*, 2023 ▸). Recently, we reported a new class of peptide nanotubes based on α-helical SLP R_3_L_12_, which forms nanotubes in aqueous solution with suitable pH, with a bilayer arrangement of the α-helices (perpendicular to the nanotube axis) such that the nanotube walls are coated with arginine (Castelletto *et al.*, 2020 ▸; Castelletto *et al.*, 2021 ▸). In a study on model lipopeptides bearing cationic lysine-rich tripeptide sequences we showed via SAXS and cryo-TEM that C_16_-Wkk (k: d-lysine) forms nanotubes coexisting with helical ribbon structures (in pH 8 aqueous solutions) (Adak *et al.*, 2024*b* ▸). The self-assembly of this lipopeptide and related ones is pH dependent, since they form micelles at lower native pH 4.6, but extended structures at pH 8 (Adak *et al.*, 2024*a* ▸; Adak *et al.*, 2024*b* ▸; Hamley *et al.*, 2024 ▸).

The modelling of SAXS data from complex self-assembled or aggregate structures presents significant challenges in describing the multi-scale structure. One approach is to perform bottom-up modelling using atomic coordinates along with a model for the molecular arrangement in the nanotube. This method has been employed in the modelling of SAXS data from tubulin microtubules, built from atomistic models of tubulin dimers positioned on a helix to build the microtubule wall (Ginsburg *et al.*, 2016 ▸; Raviv *et al.*, 2023 ▸). Such a model provides a good description of the SAXS data to high *q*. The software *D+* (Ginsburg *et al.*, 2019 ▸; Balken *et al.*, 2023 ▸) can be used to build complex hierarchical structures from subunits including microtubules and many others. Other methods such as computational reverse engineering analysis of scattering experiments (CREASE) may be used in a top-down fashion (Heil *et al.*, 2023 ▸). This approach uses a genetic algorithm with a set of ‘genes’, *i.e.* a low-dimensional feature space corresponding to a structural arrangement, and thus structure factor and particle form factor, which possesses a computed scattering profile (with optimal fitness) that most closely matches the input measured data. The method uses the Debye equation to compute the scattering from the particle feature space and/or a machine learning (ML) model to link the ‘genes’ to the scattering profile. As yet, this approach has not been applied to tubular assemblies. Simulated annealing methods using dummy atom models such as *DAMMIF* (Franke & Svergun, 2009 ▸) have been used to model microtubule-associated dimers (Svergun *et al.*, 2001 ▸; Czub *et al.*, 2025 ▸) but not tubular structures themselves. In another potential approach, constraints from SAXS or SANS can be incorporated to guide molecular dynamics simulations using Bayesian inference or the maximum entropy principle (Chen *et al.*, 2019 ▸; Chatzimagas & Hub, 2023 ▸). These methods are computationally demanding and in general require *a priori* constraints to successfully model small-angle scattering (SAS) data. Form-factor models are less computationally intensive (although may involve evaluation of multiple integrals) and are amenable to least-squares fitting using widely available SAS data fitting software such as *SASfit* (Breßler *et al.*, 2015 ▸; Kohlbrecher & Breßler, 2022 ▸), *SASViewetc.* (Hamley, 2021 ▸). Several of these programs allow incorporation of customized form factors such as those detailed below. Although form-factor fitting does benefit from prior or complementary knowledge about the self-assembled structure (for example, the morphology and associated dimensions from electron microscopy or atomic force microscopy) imaging, it does not require atomistic information.

Here we present a detailed analysis of SAS data from peptide nanotubes and related ribbon structures (Fig. 1 ▸), illustrated by data obtained for several peptide and lipopeptide systems studied in our laboratory. We provide details on several form factors that may be used to fit SAXS or SANS data from nanotubes with a defined wall structure, represented by a cross section scattering contrast (electron density for SAXS or scattering length density for SANS) profile (Fig. 1 ▸). This includes multiwall cylindrical shells as well as the Gaussian bilayer model (Pabst *et al.*, 2000 ▸), originally developed to fit SAS data for lipid bilayers but used by us for lipopeptide layer systems (lamellae, nanotapes or nanotubes). Where necessary, we discuss the incorporation of structure-factor terms for multilayer systems in the fitting of SAS data from peptide nanotubes. Certain types of peptide nanotubes coexist with, or form from (Adamcik *et al.*, 2011 ▸), wrapped helical ribbons or cochleates, and we also provide, for convenience, the form factor previously reported (Pringle & Schmidt, 1971 ▸; Hamley, 2008 ▸) for the former, as well as expressions for related cochleate structures. In addition to being useful in the analysis of SAS data from peptide nanotubes, the methods described herein will be of utility for other nanotube systems such as microtubules (Safinya *et al.*, 2016 ▸; Ginsburg *et al.*, 2016 ▸; Safinya *et al.*, 2019 ▸; Raviv *et al.*, 2023 ▸), carbon nanotubes (Wang *et al.*, 2007 ▸), hydrolyzed protein nanotubes (Graveland-Bikker *et al.*, 2006 ▸), lipid nanotubes (Wang *et al.*, 2022 ▸) and others.

## 2. Results and discussion

### 2.1. Experimental data

**Figure 2:**
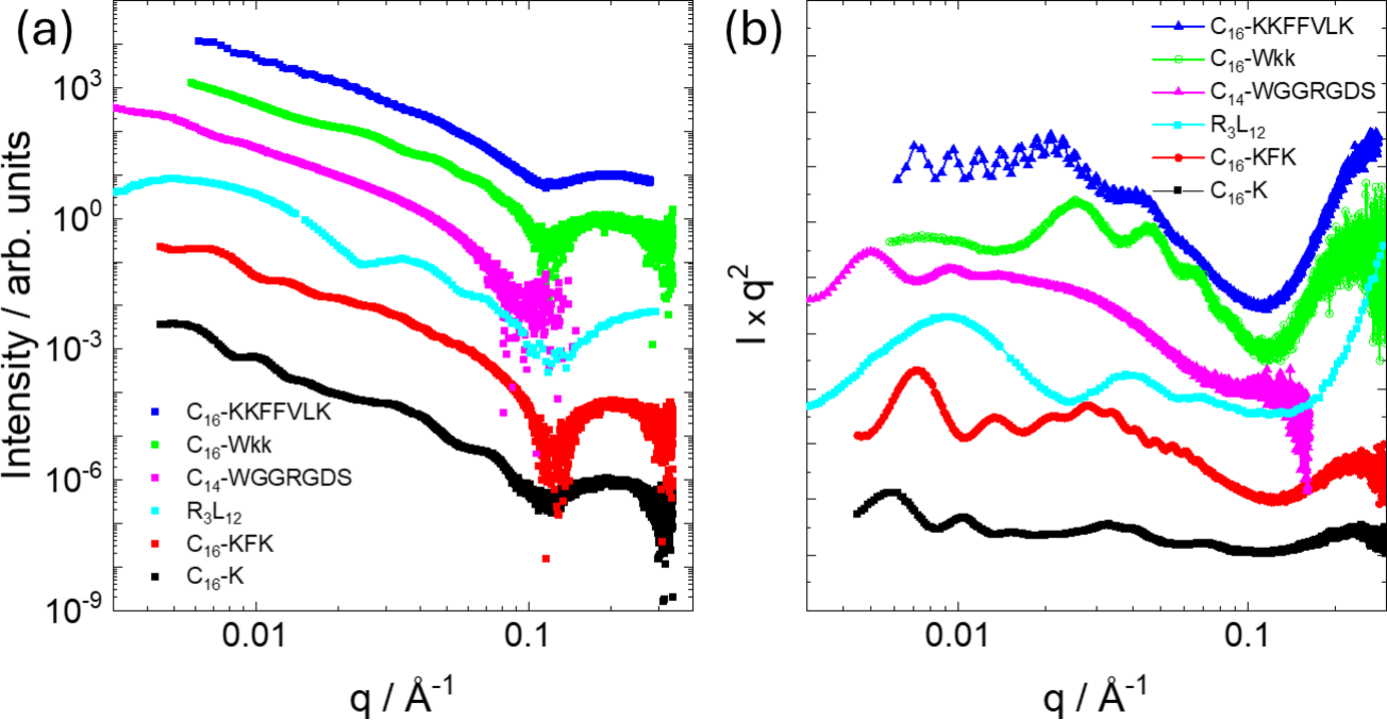
Showing examples of SAXS data from peptide nanotubes from work by our group, as indicated. All samples at 1 wt% concentration in water, unless stated otherwise. Data for C_16_-KKFFVLK (native pH) from Hamley *et al.* (2013*b* ▸). Data for C_16_-Wkk (pH 8) from Adak *et al.* (2024*b* ▸) (k denotes d-lysine). Data for C_14_-WGGRGDS (0.1 wt% in PBS, pH 7.4) from Rosa *et al.* (2023 ▸). Data for R_3_L_12_ (0.07 wt%, pH 7) from Castelletto *et al.* (2021 ▸). Data for C_16_-KFK (pH 4) and C_16_-K (pH 4) are unpublished. (*a*) Intensity versus *q* in a double-logarithmic presentation; (*b*) Kratky plots of *Iq*^2^ versus *q* (latter on a log scale). Data sets have been rescaled and shifted for comparison and ease of visualization.

High-quality SAXS data have been presented for nanotube and helical ribbon structures of many peptide and lipopeptide systems. Fig. 2 ▸ shows examples from work from our group presented in the conventional double-logarithmic representation of log *I* versus log *q* (*I*: intensity; *q*: wavenumber) and also as Kratky plots of *Iq*^2^ versus log *q*. Comparison of the plots shows that details of the form factor, especially oscillations at low *q*, are masked in the conventional double-log plots. Kratky plots provide the clearest means to distinguish such features and to compare differences between different nanotube and ribbon structures. Kratky plots are used to highlight scattering from layered structures (such plots are also termed Lorentz-corrected intensity graphs) which exhibit *q*^−2^ intensity scaling, as well as polymer coils, and to probe protein folding (Glatter & Kratky, 1982 ▸; Roe, 2000 ▸; Svergun *et al.*, 2013 ▸; Hamley, 2021 ▸).

**Figure 3:**
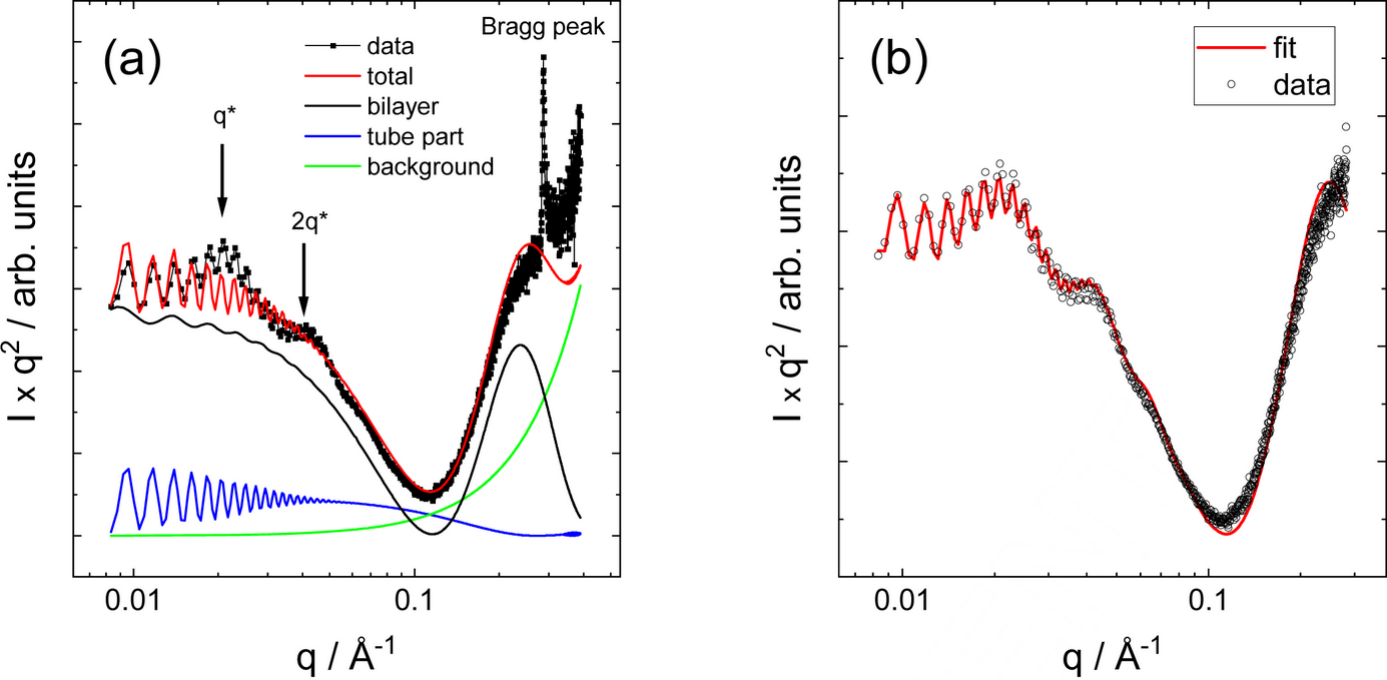
(*a*) Kratky plot of SAXS data (circles) for C_16_-KKFFVLK with separation of components from previously reported fit (lines), as described in the text, and with structure-factor peaks at q* and 2q* indicated (and a Bragg peak at high *q*). (*b*) Fit to the same data (red line) excluding the high-*q* part affected by the Bragg peak and including a lamellar structure factor, as described in the text (fit parameters in Table 1 ▸).

**Figure 4:**
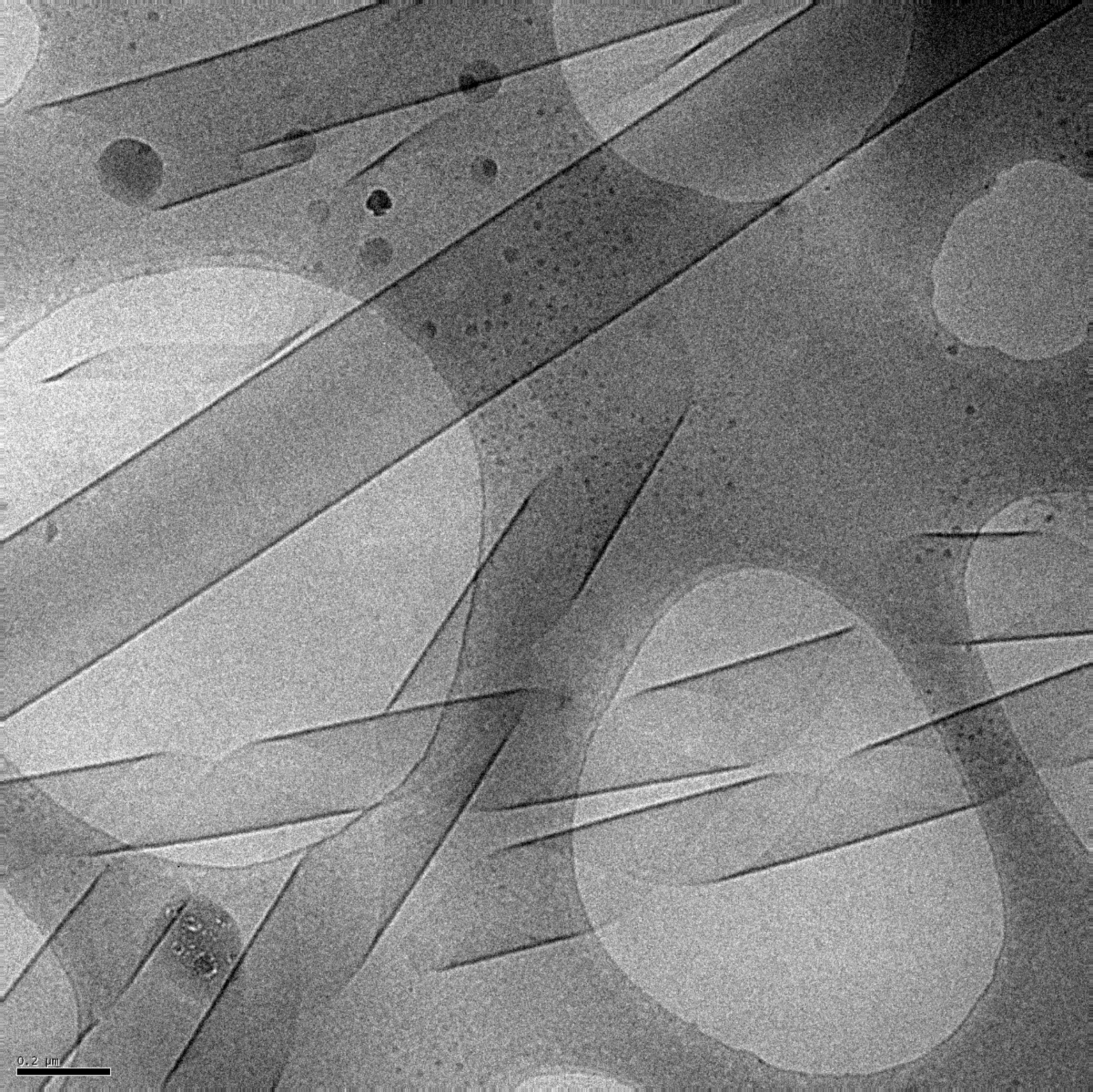
Cryo-TEM image from a 1 wt% solution of C_16_-KKFFVLK.

The SAXS data for C_16_-KKFFVLK in Fig. 2 ▸ show high-frequency fringes at low *q* which arise from interference scattering from the nanotube walls, the nanotube having a large radius *R* = 1380 Å as reported previously (Hamley *et al.*, 2013*b* ▸). The data were fitted using the sum of hollow cylinder + Gaussian bilayer (Fig. 1 ▸) form factors. The data and components of the original fit are shown in Fig. 3 ▸. The former allows for the nanotube scattering (low *q*) and the latter for the structure within the nanotube walls (high *q*). The Gaussian bilayer form factor was developed to describe the form factor from lipid bilayers resulting from a density profile (here: electron density) represented as a sum of three Gaussian functions describing the electron density of the lipid core (with reduced electron density, typically lower than that of the aqueous solvent) and the headgroup regions (with enhanced positive electron density) (Pabst *et al.*, 2000 ▸). As discussed below in the theory section, this model is an approximation to the full expression for the form factor of a nanotube with structured cross-sectional electron-density profile. The fit in Fig. 3 ▸(*a*) describes the period of the low-*q* fringes well, and also the shape of the profile at high *q* which is due to the structure within the nanotube wall represented as a Gaussian bilayer [the broad weak oscillations at low *q* in this component, black line in Fig. 3 ▸(*a*), are due to the finite width of this component, although this was not varied during the fit and does not influence the present discussion]. However, a careful inspection of the data in a Kratky plot reveals the modulation of the low-*q* intensity, most likely due to structure-factor peaks as shown at q* = 0.02 Å^−1^ and 2q* = 0.04 Å^−1^, indicating a lamellar-type structure factor with period 284 Å. These data were re-fitted using a modified model that incorporates a lamellar structure factor [Caillé type (Caillé, 1972 ▸)] for the bilayer component of the fit. A good fit is obtained with this model, as shown in Fig. 3 ▸(*b*), with fit parameters listed in Table 1 ▸. The presence of a (large period) lamellar structure is consistent with the presence of helical ribbon and tape structures in the cryo-TEM images (Hamley *et al.*, 2013*b* ▸), such as that shown in Fig. 4 ▸. The data in Fig. 3 ▸(*b*) were fitted using *SASfit* (Breßler *et al.*, 2015 ▸; Kohlbrecher & Breßler, 2022 ▸), which performs χ^2^ minimization with experimental error bar weighting of intensity.

**Figure 5:**
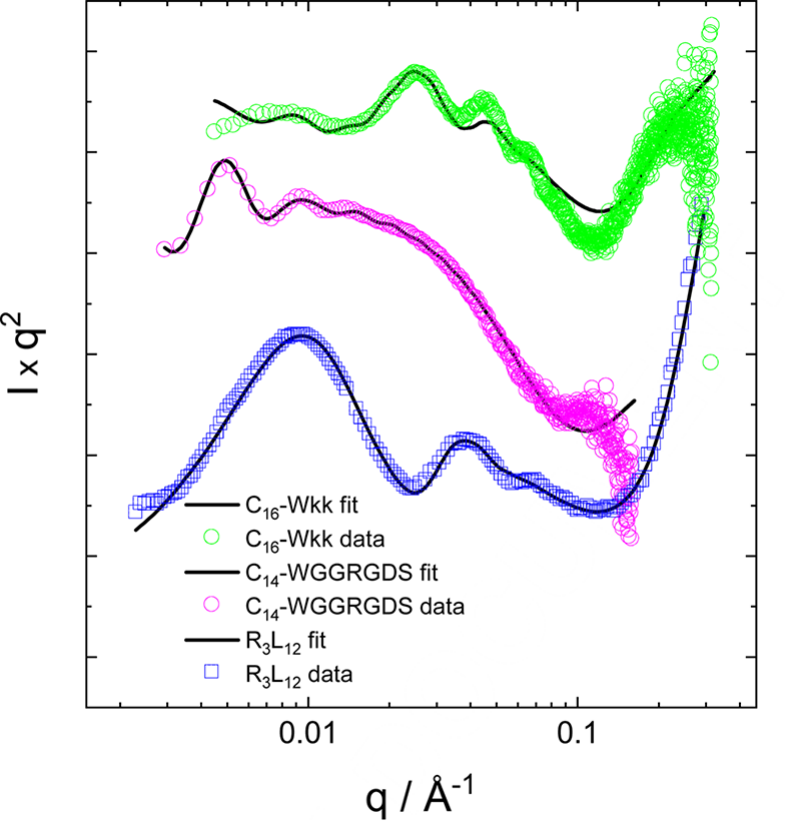
SAXS data (same as in Fig. 2 ▸, open symbols) with fits (solid lines) for C_16_-Wkk (Adak *et al.*, 2024*b* ▸), C_14_-WGGRGDS (Rosa *et al.*, 2023 ▸) and R_3_L_12_ (Castelletto *et al.*, 2021 ▸). Data sets have been rescaled and shifted for comparison (and only every third data point plotted for the C_16_-Wkk and C_14_-WGGRGDS data) and ease of visualization.

Consideration of structure-factor effects is not necessary to fit the example data in Fig. 2 ▸ for C_16_-Wkk (Adak *et al.*, 2024*b* ▸), C_14_-WGGRGDS (Rosa *et al.*, 2023 ▸) or R_3_L_12_ (Castelletto *et al.*, 2021 ▸). The published fits were improved by using the combined hollow cylinder + Gaussian bilayer form factor. The fits are good quality, as evident from Fig. 5 ▸ and the fit parameters are listed in Table 1 ▸.

**Figure 6:**
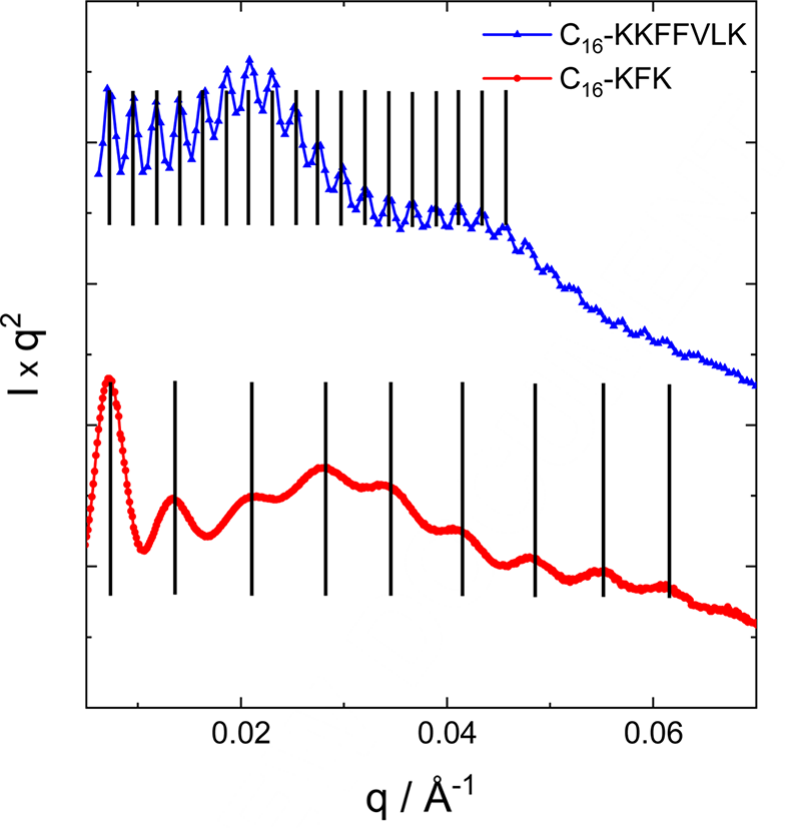
SAXS data (expanded low-*q* region on linear *q* scale) for C_16_-KFK (1 wt%, pH 4) showing aperiodicity of form-factor oscillations in contrast to C_16_-KKFFVLK (1 wt%, pH native).

**Figure 7:**
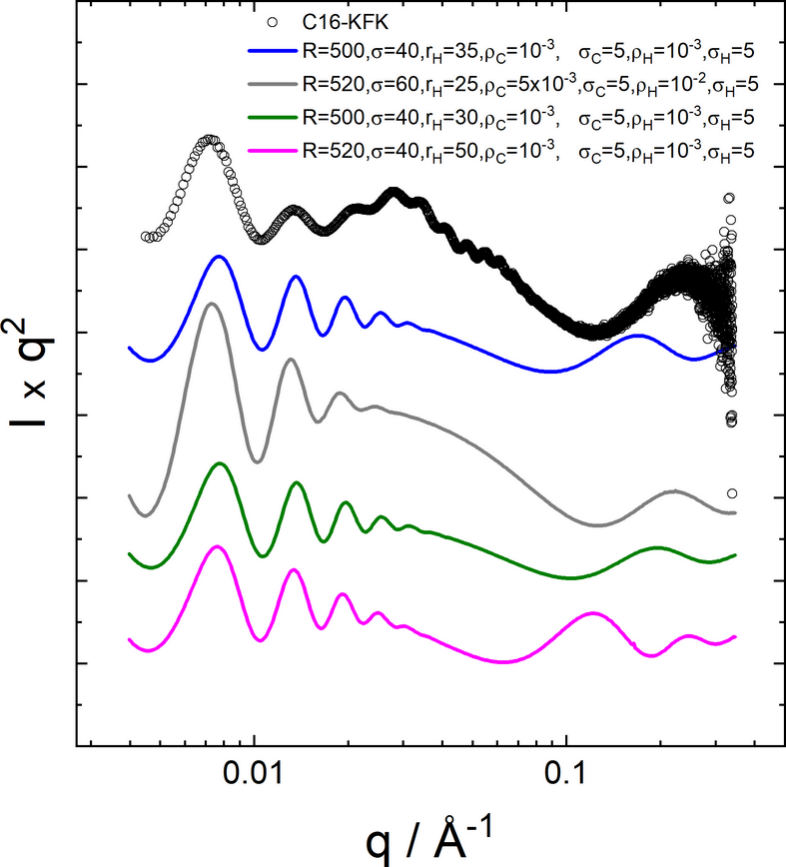
Examples of form factors calculated using the nanotube with Gaussian bilayer wall structure (Fig. 1 ▸) via equations (7)–(9), compared with measured data (open symbols) for C_16_-KFK (same data and conditions as Fig. 2 ▸). Parameters [Fig. 1 ▸ and equation (9)]: *R* nanotube radius (to centre of wall, Å) with Gaussian polydispersity σ, *r*_H_ half bilayer thickness in Å, σ_C_, ρ_C_ central Gaussian width (Å) and scattering density (arbitrary units), σ_H_, ρ_H_ outer (headgroup) Gaussian width (Å) and scattering density (arbitrary units), *L* = 3000 Å fixed length of nanotube. Data sets have been rescaled and shifted for comparison and ease of visualization.

**Figure 8:**
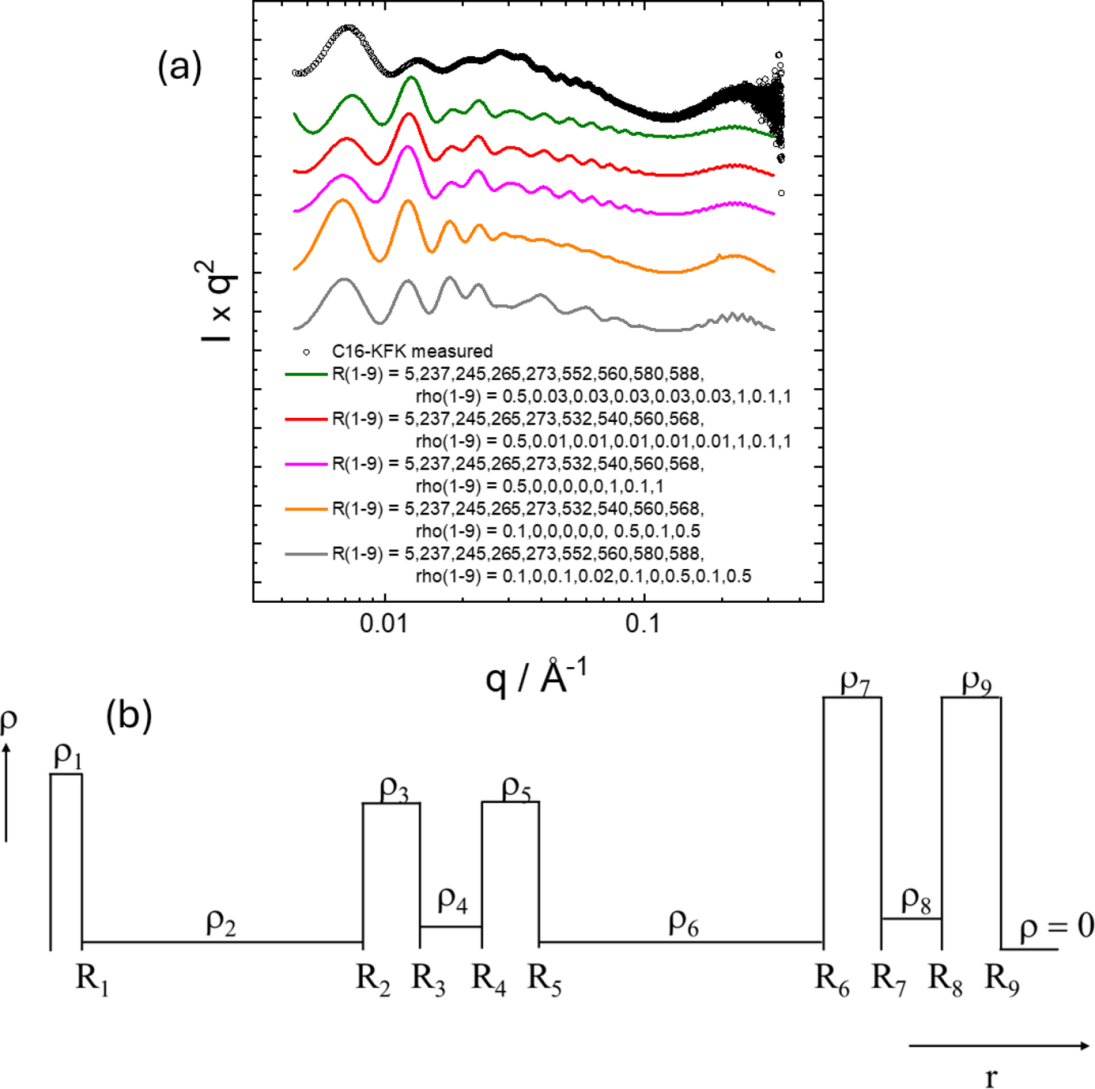
(*a*) Examples of form factors calculated for a nanotube with multishell structure [slab mode, equations (10) and (11)], compared with measured data (open symbols) for C_16_-KFK (same as Fig. 2 ▸). In this series of calculations a thin central core was included in order to introduce high-frequency fringes at high *q*, as observed in the experimental data. For all calculations (radii in ångström and scattering densities in arbitrary units indicated) the nanotube length was *L* = 2000 Å and the radius polydispersity was σ = 40 Å. Data sets have been rescaled and shifted for comparison and ease of visualization. (*b*) Scheme of slab cross section scattering density profile for the calculations.

The data for C_16_-KFK and C_16_-K in Fig. 2 ▸ have more complex features at low *q* with aperiodic form-factor maxima and intensity increases reminiscent of broad structure-factor peaks (compare with the data for C_16_-KKFFVLK in Fig. 2 ▸). The aperiodicity in the form-factor oscillations for C_16_-KFK shown in Fig. 6 ▸ is contrasted with the precise periodicity in the data for C_16_-KKFFVLK. In the former case, the periodicity of the oscillations is disrupted around the broad local maximum around *q* = 0.03 Å^−1^, which may be due to the structure factor or a broad form-factor peak. The corresponding periodicity is 210 Å, which does not appear to relate to a structure-factor repeat distance since the nanotube radius is around 500 Å (Figs. 7 and 8) and seems too large to correspond to an internal periodicity. As described below, efforts were made to model these data on the basis of form-factor models for cylindrical slab structures or Gaussian bilayers via equations (7)–(9) (*i.e.* without the approximation decoupling the nanotube and Gaussian bilayer terms). However, these models were not able to describe satisfactorily the full aperiodic *q*-dependent scattering observed (Fig. 6 ▸). It is believed that these SAXS intensity profiles reflect contributions from multiple nano­structures in the system, including nanotubes, helical ribbons and cochleate (scroll) structures. These are in fact apparent from inspection of cryo-TEM images, which resemble that shown in Fig. 4 ▸ (Gao *et al.*, 2019 ▸; Hamley *et al.*, 2025 ▸).

### 2.2. SAXS data – general theory

The intensity as a function of wavenumber *q* for an isotropic solution of particles is written generally as the product of a form factor *P*(*q*) due to the internal structure of the particle and the structure factor *S*(*q*) depending on inter-particle interactions (Pedersen, 1997 ▸; Pedersen, 2002 ▸; Hamley, 2021 ▸):
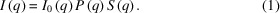
Here *I*_0_ is a normalization factor to put the intensity on an absolute scale (Hamley, 2021 ▸; Pozza & Bonneté, 2023 ▸; Hamley & Castelletto, 2024 ▸). In the following, the interactions between nanotubes are not considered [*S*(*q*) = 1], which is a reasonable approximation in dilute solution. However, in some cases we have introduced a lamellar structure factor for possible multilamellar structures (twisted tapes, helical ribbons, cochleates) which coexist with nanotubes in some systems. Structure-factor effects due to inter-layer correlations in the walls of nanotube structures may lead to peaks typically in the wide-angle region, considering the packing for example of antiparallel bilayers of peptides or lipopeptides; indeed, this is observed in experimental XRD or wide-angle X-ray scattering data (Valéry *et al.*, 2003 ▸; Mehta *et al.*, 2008 ▸; Childers *et al.*, 2009 ▸; Castelletto *et al.*, 2010 ▸; Hamley *et al.*, 2013*b* ▸; Valéry *et al.*, 2015 ▸; Gao *et al.*, 2019 ▸; Narayanan *et al.*, 2021*a* ▸; Narayanan *et al.*, 2021*b* ▸; McCourt *et al.*, 2022 ▸). This is not discussed further herein since the focus is on SAXS form-factor analysis.

The form-factor intensity for an isotropic solution of polydisperse particles can be written
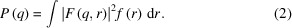
Here *F*(*q*,*r*) is the form-factor amplitude and *f*(*r*) is the polydispersity in size, for example a Gaussian function (width σ, centre *R*):
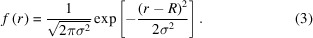


For a long cylindrical structure with length *L* >> *R* (the average radius), the form-factor intensity can be written as the product of scattering dependent on *L* (axial scattering) and that of the cross section (Porod, 1982 ▸):
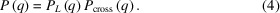
Here the axial form-factor intensity is (Porod, 1982 ▸)
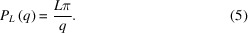


Various models for the cross section form-factor intensity *P*_cross_(*q*) are discussed below. For the simple case of a uniform (scattering density ρ) cylinder of radius *R*, it is given by (Porod, 1982 ▸)
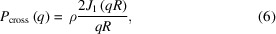
where *J*_1_ is a first-order Bessel function.

### 2.3. Modelling – general cross section form factor

For a cylindrical structure, in the most general case [without decoupling of axial and cross section terms as in equation (4)] the form-factor intensity takes the form (Fournet, 1951 ▸; Guinier & Fournet, 1955 ▸; Porod, 1982 ▸)
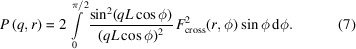
Here, the cross section amplitude term is given by (Fournet, 1951 ▸)
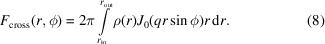
The integral extends over the inner and outer radii of the nanotube (*i.e.* over the nanotube wall thickness) and *J*_0_ denotes a zeroth-order Bessel function.

For the Gaussian bilayer model, the electron density ρ(*r*) is represented as a sum of three Gaussian functions (Pabst *et al.*, 2000 ▸):
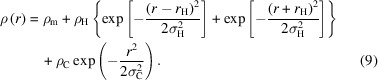
The parameters are defined in Fig. 1 ▸. The limits for the integral in equation (8) are here taken to be *r*_in_ = *R* − 2*r*_H_, *r*_out_ = *R* + 2*r*_H_, where *r*_H_ is half the bilayer thickness. Examples of SAXS form-factor profiles calculated with the full Gaussian bilayer nanotube form factor using equations (7)–(9) are presented in Fig. 7 ▸, in comparison with measured data for C_16_-KFK. The models, in particular the example plotted as a grey line, describe the overall shape of the profile, as well as the position of the oscillations at low *q*. However, the models do not account for the higher-frequency oscillations at intermediate *q* or the aperiodicity of these fringes around the broad maximum at *q* = 0.03 Å^−1^, discussed in more detail in Section 2.2. Further modelling was undertaken using a slab model for multishell nanotubes, as described in the following.

### 2.4. Modelling – slab model for multiwall nanotubes

The cross section form-factor amplitude for a tube containing multiple concentric cylindrical shells (as in a multiwall nanotube), each of radius *R*_*i*_ and scattering density ρ_*i*_, has been provided by several authors (Livsey, 1987 ▸; Teixeira *et al.*, 2010 ▸; Paineau *et al.*, 2016 ▸; Landman *et al.*, 2018 ▸; Komarova *et al.*, 2025 ▸). Here we follow Teixeira *et al.* (2010 ▸) and define the form-factor amplitude for an *N* concentric cylindrical shell system in the following form:
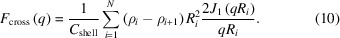
Here *J*_1_ is a first-order Bessel function and *C*_shell_ is a normalization factor (Teixeira *et al.*, 2010 ▸):
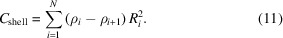


Examples of SAXS form-factor profiles calculated using a multishell form factor (multiple slab density profile) are presented in Fig. 8 ▸. Despite exploring an extensive parameter range (much beyond the examples in Fig. 8 ▸), it was not possible to fit the measured form-factor data for C_16_-KFK. This is ascribed to the presence of multiple coexisting nanostructures in this system (confirmed by cryo-TEM) including nanotubes, cochleates and helical ribbons (Hamley *et al.*, 2025 ▸). Nonetheless, this form factor is useful for defined multishell nanostructures (Teixeira *et al.*, 2010 ▸).

### 2.5. Modelling – form factor of ribbons

For convenient reference, form factors are provided below for helical ribbon and cochleate structures. These will be useful for systems known to form such structures. For the lipopeptides studied to date by us, such as C_16_-KKFFVLK, C_16_-K and C_16_-KWK discussed above, these structures are observed (by cryo-TEM) in coexistence with other structures. Therefore, to date, these form factors have not been applicable to lipopeptide systems forming these structures in isolation.

The form factor for helical ribbons can be obtained from the expressions derived for helical ribbons (Pringle & Schmidt, 1971 ▸). They provide a general form factor for two helical ribbons offset by a defined angle. The simplified expression for a single helical tape of pitch *p* and outer radius *R* and inner radius *aR* is written (Teixeira *et al.*, 2010 ▸)
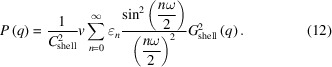
Here, *C*_shell_ is given by equation (11), ɛ_0_ = 1 and ɛ_*n*_ = 2 for *n* ≥ 1, ω is the angular range of a ribbon in the cross section plane, and (summing over the shells with scattering density ρ_*i*_ and radius *R*_*i*_) (Teixeira *et al.*, 2010 ▸)
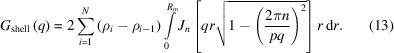
The infinite sum in equation (12) is replaced by a finite sum (to *N* shells) since at a certain value of *q* only layer lines with *n* ≤ *Pq*/(2π) contribute to the sum (Teixeira *et al.*, 2010 ▸).

**Figure 9:**
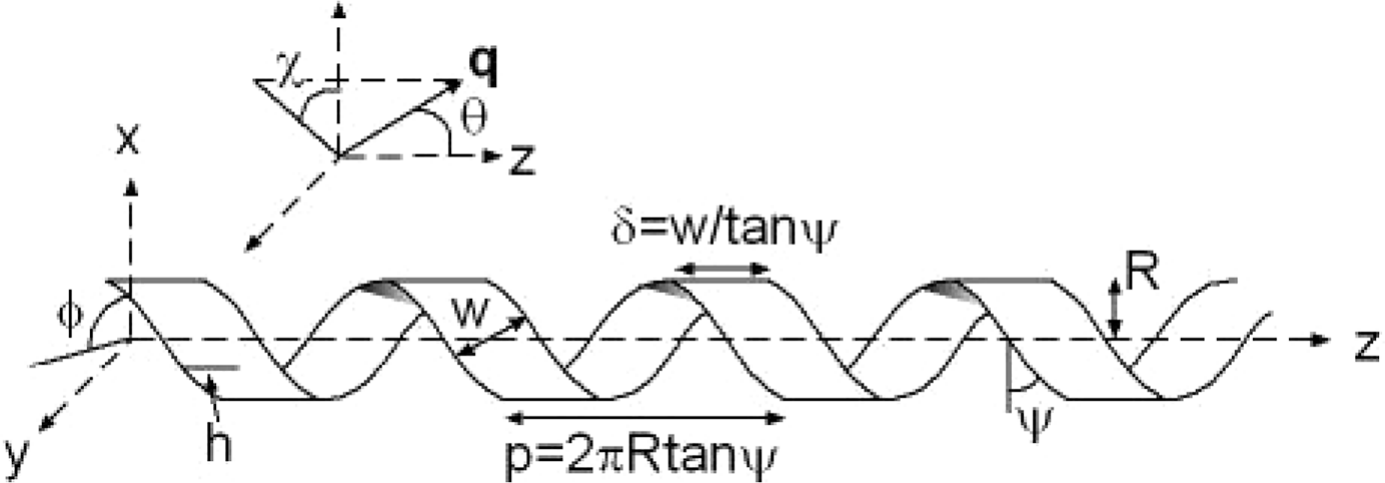
Single infinitesimally thick helical ribbon showing definition of variables.

A form factor has also been derived (Hamley, 2008 ▸) for a helical ribbon (Fig. 9 ▸) of infinitesimal thickness and uniform scattering density ρ with coordinates of a surface point (*R* cos ϕ, *R* sin ϕ, *R*ϕ tan ψ + *h*). The variables are defined as *h*, a coordinate along the ribbon axis *z*, ϕ the rotation angle around *z* and ψ a helical twist angle (Fig. 9 ▸). The form-factor amplitude for the aligned ribbon using Cartesian coordinates is then
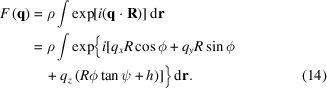
Here **q** is the wavevector (with angles with respect to the reference frame shown in Fig. 9 ▸). The ribbon is a two-dimensional object in three-dimensional space, and the integral can be evaluated using polar coordinates (ϕ, *h*). Then d**r** = dϕ d*h*. Additionally, using polar coordinates in the same ribbon fixed-axis system for **q** = (*q* sin θ cos χ, *q* sin θ sin χ, *q* cos θ) we obtain
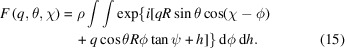
This integral can be evaluated by taking the integral over *h*, for a single turn of the ribbon, and allowing for centrosymmetry (Hamley, 2008 ▸):
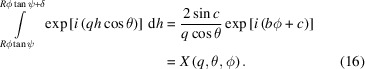
Here *b* = *q* cos θ*R* tan ψ and 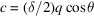.

The amplitude form factor for *m* repeats of a helical ribbon in a fixed orientation is then given by
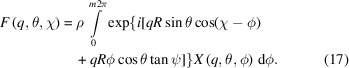
This is the amplitude form factor of a single helical ribbon in a fixed orientation (ϕ, ψ, θ). As discussed previously (Pringle & Schmidt, 1971 ▸; Hamley, 2008 ▸), the scattering in the (*q*_*x*_, *q*_*z*_) and (*q*_*y*_, *q*_*z*_) planes is dominated by intensity in layer lines at *q*_*z*_ = *n*π/*p*, the intensity being more concentrated as *m*, *i.e.* the number of helical repeats, increases. The integral in equation (15) can then be evaluated, leading to the approximate expression
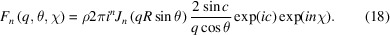
This is the form factor derived for a helix (Cochran *et al.*, 1952 ▸) convoluted with the term 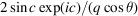 contained in *X*(*q*, θ, ϕ) which results from the width of the ribbon.

The isotropically averaged form factor takes the general form
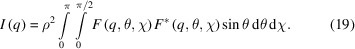
It can be evaluated by assuming that the intensity is totally concentrated on the layer lines, and in this case we obtain
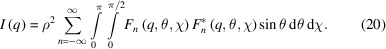
In practice, the sum only needs to extend over the layer lines in the *q* range accessed, which can be readily determined from the position of the *n*th layer line at *q*_*z*_ = *n*π/*p*.

Equations (18) and (20) are valid for *p* ≡ 2π*b* > 2δ. In the limiting case *p* = 2δ, we have an infinitesimal cylindrical tube (length *P*) for which *X*(*q*, θ, ϕ) = 1 and the isotropically averaged form factor is given exactly by (Hamley, 2008 ▸)
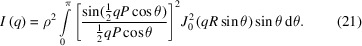


**Figure 10:**
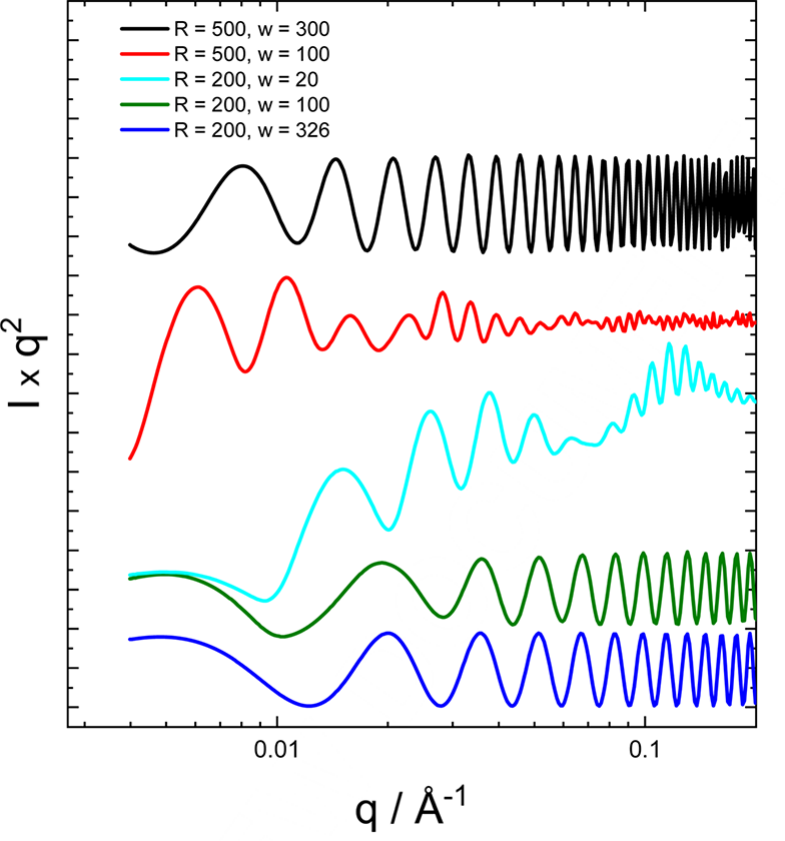
Examples of form factors for monodisperse helical ribbons calculated using equation (20) with parameter radius *R* (Å), ribbon width *w* (Å), scattering density ρ = 1 (arbitrary units) and helix angle ψ = 27°, and with the integral in equation (17) with *m* = 10. The case *R* = 200 Å, *w* = 326 Å, ψ = 27° corresponds approximately to nanotubes (full wrapping) since under these conditions δ ≃ *p* = 2π*R* tan^2^ ψ (Fig. 9 ▸). Data sets have been rescaled and shifted for comparison and ease of visualization.

Rather than taking the approximation of intensity concentrated on layer lines as in our previous report (Hamley, 2008 ▸), we evaluated equation (19) by direct integration and plot several examples of such calculations in Fig. 10 ▸. This shows the expected influence of ribbon radius (increasing this leads to more closely spaced form-factor peaks) as well as the effect of variation of ribbon width *w* and angle ψ. The limiting behaviour of the form factor of a nanotube [equation (21)] is recovered in the case that the ribbon width δ is equal to its pitch *p*. McCourt *et al.* (2022 ▸) present the form factor for helical ribbons in a different form to equation (19) using the extended Pringle–Schmidt form factor obtained by Teixeira *et al.* (2010 ▸) and use it to fit SAXS data from C_*n*_-K lipopeptides with *n* = 12, 14, 16 at different pH values in aqueous solution.

**Figure 11:**
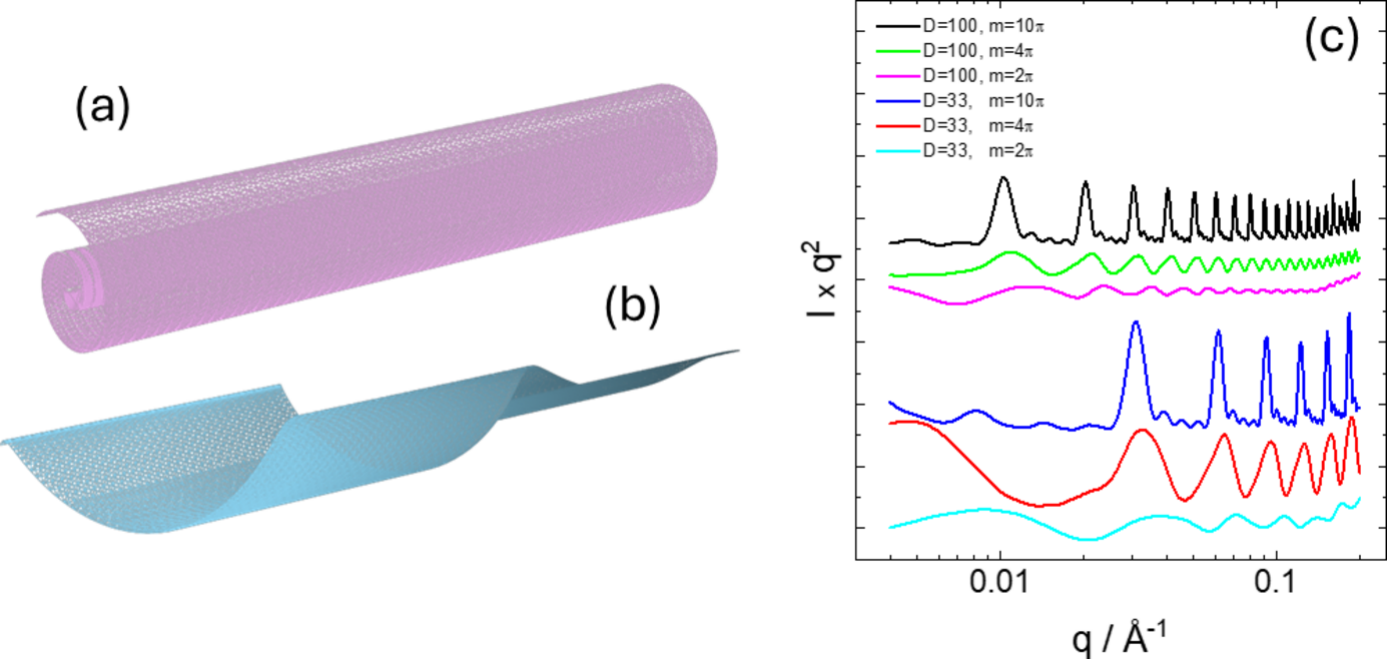
(*a*) Cochleate (carpet roll, Archimedean spiral cross section) structure, (*b*) helical cochleate structure and (*c*) examples of form-factor calculations for cochleate, all with ρ = 1 and δ = 2000. Data sets have been rescaled and shifted for comparison and ease of visualization.

For a cochleate (scroll or rolled-up carpet) structure [Fig. 11 ▸(*a*)] (infinitesimally thin sheet), the cross section of which is an Archimedean spiral, the coordinates in polar form of the surface are (*D*ϕ cos ϕ, *D*ϕ sin ϕ, *z*). The form factor can be derived analogously to equations (14) to (17) but leading to an amplitude form factor
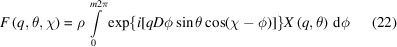
with 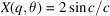 and 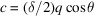.

Examples of form factors computed using equation (22) are shown in Fig. 11 ▸(*c*). The sharp peaks arise from the rolled layer (with repeat 2π*D*), the sharpness increasing with the number of turns of the spiral [parameter *m* in equation (22)].

For helical cochleates [Fig. 11 ▸(*b*)] with polar coordinates (*D*ϕ cos ϕ, *D*ϕ sin ϕ, *z* + *p*ϕ), where *p* is the pitch, the form factor is similarly derived as
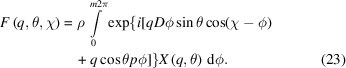
This gives form factors with similar features to those shown in Fig. 11 ▸(*c*) for non-helical ribbons, *i.e.* for sufficiently large *m* there are ‘Bragg-like’ peaks due to the spiral ‘layer’ repeats.

## 3. Conclusions

Peptide nanotubes have a diversity of structures, reflecting the distinct modes of assembly such as wrapping of β-sheet ribbons, packing of SLPs and others. This leads to distinct form factors in SAS data, due to differences in nanotube radius and especially structure within the nanotube wall. These features are clearly apparent when the data are plotted in ‘Kratky’ form of *Iq*^2^ versus *q*. Selected examples discussed here show considerable differences in the detailed features. These can be described using models for the electron-density profile across the nanotube wall via slab models for concentric shells in multiwall nanotubes, or other models such as the Gaussian bilayer model which we have used to fit data from several lipopeptide and peptide systems, as discussed above. This can be implemented explicitly within the calculation of the form factor from the general expressions given as equations (7) and (8), although this is computationally intensive since, allowing for polydispersity, it requires the evaluation of a triple integral. It is more convenient to treat the Gaussian bilayer contribution in a ‘convolution’ with the hollow cylinder scattering from the nanotube (*i.e.* the form factor is represented as a sum of these terms). This can be implemented within software to fit SAS data such as *SASfit* (Breßler *et al.*, 2015 ▸; Kohlbrecher & Breßler, 2022 ▸), which enables efficient least-squares fitting of experimental data with such models. We also present re-analysis of the well defined SAXS data for C_16_-KKFFVLK, which reveals structure-factor features, fitted using the Caillé model for fluctuating lamellar structures with diffuse structure-factor scattering. As noted in the *Introduction*, other groups have also considered structure-factor contributions in the scattering from peptide nanotube systems, including lamellar or hexagonal structure factors (Valéry *et al.*, 2003 ▸; Lu *et al.*, 2007 ▸; Cenker *et al.*, 2011 ▸; Siegl *et al.*, 2021 ▸).

The form factor for helical ribbon structures [reported for C_16_-KKFFVLK, Fig. 3 ▸, and other peptide systems (Siegl *et al.*, 2021 ▸; Wang *et al.*, 2021 ▸)] is here evaluated directly [via a triple integral, equations (17) and (19)] without the previous approximations concerning the concentration of scattering on layer lines (Pringle & Schmidt, 1971 ▸; Teixeira *et al.*, 2010 ▸; Hamley, 2008 ▸). As exemplified by data for C_16_-KFK and C_16_-K, the scattering for some systems is complicated by the presence of coexisting structures and here the complementary use of electron microscopy or other microscopy methods (atomic force microscopy) along with SAS is desirable in resolving this, although certain features in the SAS data such as aperiodic form-factor oscillations point to the presence of species other than simple nanotubes. This includes cochleates (Gao *et al.*, 2019 ▸; McCourt *et al.*, 2022 ▸), and here we provide expressions for the form factors of such structures. The analysis methods presented here are expected to be useful as new examples and classes of peptide nanotube structures are uncovered, and for other nanotube-forming materials.

## Figures and Tables

**Table 1:** Parameters extracted from the fitting of the SAXS data for the data in Figs. 3 ▸ and 5 ▸ Key. *Gaussian bilayer*: layer thickness *t* (Gaussian polydispersity Δ*t*), scattering contrast of outer (headgroup) layers ρ_H_ and core (lipid chain) layer ρ_C_, Gaussian widths σ_C_ and σ_H_ of core and headgroup layers, respectively, *D* diameter (width) of layer system (when *D* >> *t* as here, it acts as a scaling parameter for the form factor). *Caillé structure factor*: number of layers *N*, layer spacing *d*, Caille parameter η, additional diffuse scattering *ν*. *Long cylindrical shell*: *R* core radius (Gaussian polydispersity Δ*t*), *s* shell thickness, scattering contrasts of core ρ_core_, shell ρ_shell_ and solvent ρ_solv_, *L* length. *Background*: constant background, *C*. Weightings for two-component form factors, *w*_1_, *w*_2_. Data fitted using the software *SASfit* (Breßler *et al.*, 2015 ▸; Kohlbrecher & Breßler, 2022 ▸).

	1 wt% C_16_-KKFFVLK (Fig. 3) (Hamley *et al.*, 2013*b* ▸)	1 wt% C_16_-KKFFVLK (Fig. 3)	1 wt% C_16_-Wkk (Fig. 5)	0.1 wt% C_14_-WGGRGDS (Fig. 5)	0.07 wt% R_3_L_12_ (Fig. 5)
*w*_1_	2	0.034	6.097	0.816	N/A
*t* ± Δ*t* (Å)	23.0 ± 1.3	23.0 ± 1.3†	17.4 ± 1.6	20.0 ± 5.0	24.0 ± 2.0
ρ_H_	1.06 × 10^−2^	6.1 × 10^−3^	1.23 ×10^−7^	1.59 × 10^−6^	6.42 × 10^−7^
σ_H_ (Å)	4.3	4.3†	6.2	5	6.4
ρ_C_	−1.02 × 10^−2^	−5.96 × 10^−3^	−1.45 × 10^−7^	−1.53 × 10^−6^	−9.82 × 10^−8^
σ_C_ (Å)	1.9	1.9†	4.3	5	10.0
*D* (Å)†	130	403	1276	1170	700
*N* †	–	2	–	–	–
*d* (Å)	–	284	–	–	–
η	–	0.063	–	–	–
ν	–	18.3	–	–	–
*w*_2_	1.4	0.015	0.354	0.545	0.973
*R* (Å)	1400 ± 27	1400 ± 27†	125 ± 20	725 ± 160	73 ± 3
*s* (Å)	23.0	23.0†	26.5	26.5	28.8
ρ_core_	1.10 × 10^−4^	1.01 × 10^−4^	9.94 × 10^−8^	1.11 × 10^−7^	2.24 × 10^−6^
ρ_shell_	−7.60 × 10^−4^	−4.64 × 10^−5^	−1.39 × 10^−7^	2.46 × 10^−7^	1.48 × 10^−5^
ρ_solv_†	1.10 × 10^−4^	1.10 × 10^−4^†	6.10 × 10^−8^	1.00 × 10^−7^	2.10 × 10^−6^
*L* †	300	6.31 × 10^6^	1505	1212	505
*C*	4.00	2.77	2.89 × 10^−3^	−0.62 × 10^−2^	6 × 10^−4^

† Fixed parameter.
